# Crystal structure of tris­(phenyl­seleno­lato-κ*Se*)tris­(tetra­hydro­furan-κ*O*)thulium(III)

**DOI:** 10.1107/S1600536814023733

**Published:** 2014-10-31

**Authors:** Esther M. Takaluoma, Raija Oilunkaniemi, Christian W. Lehmann, Risto S. Laitinen

**Affiliations:** a Department of Chemistry, PO Box 3000, FI-90014 University of Oulu, Finland; b Max-Planck-Institut für Kohlenforschung, Kaiser-Wilhelm-Platz 1, D-45470 Mülheim an der Ruhr, Germany

**Keywords:** crystal structure, thulium complex, phenyl­seleno­late ligand

## Abstract

In the title compound, [Tm(C_6_H_5_Se)_3_(C_4_H_8_O)_3_], the Tm^III^ atom lies on a threefold rotation axis and is coordinated by three phenyl­seleno­late ligands and three tetra­hydro­furan ligands leading to a distorted *fac*-octa­hedral coordination environment. The Tm—Se and Tm—O bond lengths are 2.7692 (17) and 2.345 (10) Å, respectively, and the bond angles are 91.32 (6)° for Se—Tm—Se, 92.6 (2) and 94.4 (2)° for Se—Tm—O, and 81.2 (3)° for O—Tm—O. In the crystal, the discrete complexes are linked by van der Waals inter­actions only. The crystal was refined as a non-merohedral twin (ratio = 0.65:0.35).

## Related literature   

For the synthesis of the title compound, see: Lee *et al.* (1998 ▶). For the crystal structures of the isotypic compounds [Er(SePh)_3_(THF)_3_] and [Yb(SePh)_3_(THF)_3_], see: Lee *et al.* (1998 ▶); Geissinger & Magull (1995 ▶). For a binuclear seleno­late complex of thulium, see: Lee *et al.* (1995 ▶).
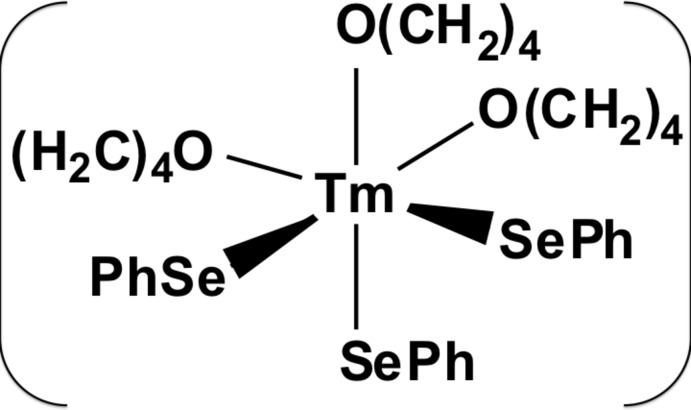


## Experimental   

### Crystal data  

[Tm(C_6_H_5_Se)_3_(C_4_H_8_O)_3_]
*M* *_r_* = 853.42
Trigonal, 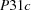

*a* = 15.277 (2) Å
*c* = 7.8708 (16) Å
*V* = 1590.9 (6) Å^3^
*Z* = 2
Mo *K*α radiation
μ = 6.25 mm^−1^
*T* = 120 K
0.40 × 0.20 × 0.10 mm

### Data collection  

Bruker–Nonius KappaCCD diffractometer
Absorption correction: multi-scan (*XPREP* in *SHELXTL*; Sheldrick, 2008 ▶) *T*_min_ = 0.189, *T*_max_ = 0.574
5660 measured reflections
2151 independent reflections
2053 reflections with *I* > 2σ(*I*)
*R*_int_ = 0.053

### Refinement  

*R*[*F* ^2^ > 2σ(*F* ^2^)] = 0.038
*wR*(*F* ^2^) = 0.087
*S* = 1.10
2151 reflections
127 parameters
9 restraints
H-atom parameters constrained
Δρ_max_ = 0.76 e Å^−3^
Δρ_min_ = −1.43 e Å^−3^
Absolute structure: Flack *x* determined using 812 quotients [(*I* ^+^)−(*I* ^−^)]/[(*I* ^+^)+(*I* ^−^)] (Parsons & Flack, 2004 ▶)
Absolute structure parameter: −0.03 (3)

### 

Data collection: *COLLECT* (Bruker, 2008 ▶); cell refinement: *DENZO-SMN* (Otwinowski & Minor, 1997 ▶); data reduction: *DENZO-SMN*; program(s) used to solve structure: *SIR92* (Altomare *et al.*, 1993 ▶); program(s) used to refine structure: *SHELXL2013* (Sheldrick, 2008 ▶); molecular graphics: *DIAMOND* (Brandenburg, 2006 ▶); software used to prepare material for publication: *WinGX* (Farrugia, 2012 ▶).

## Supplementary Material

Crystal structure: contains datablock(s) I, global. DOI: 10.1107/S1600536814023733/su5009sup1.cif

Structure factors: contains datablock(s) I. DOI: 10.1107/S1600536814023733/su5009Isup2.hkl

**Fig. 1.**
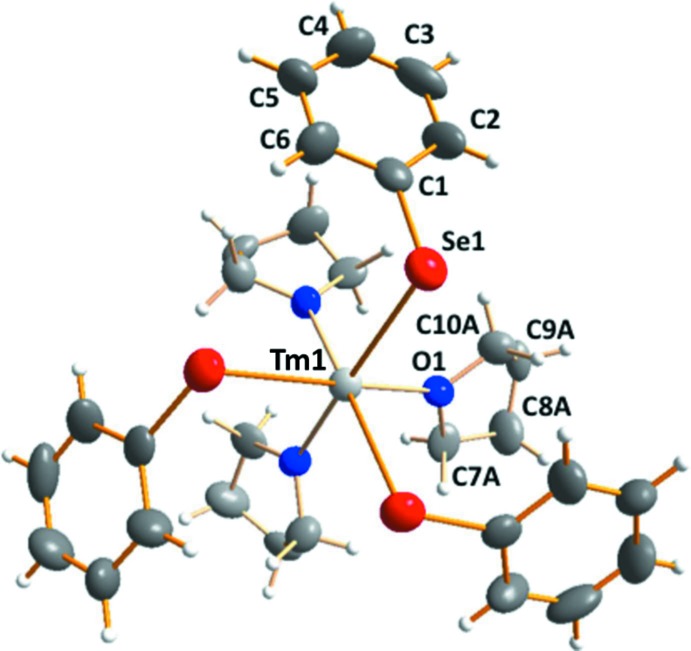
The molecular structure of the title compound, with atom labelling. Displacement ellipsoids are drawn at the 50% probability level. Only the more abundant orientation of the disordered THF ligands is shown (symmetry codes: (i) x, y, z; (ii) -y, x-y, z; (iii) -x + y, -x, z; (iv) y, x, z + 1/2; (v) x-y, -y, z + 1/2; (vi) -x, -x + y, z + 1/2.

**Fig. 2.**
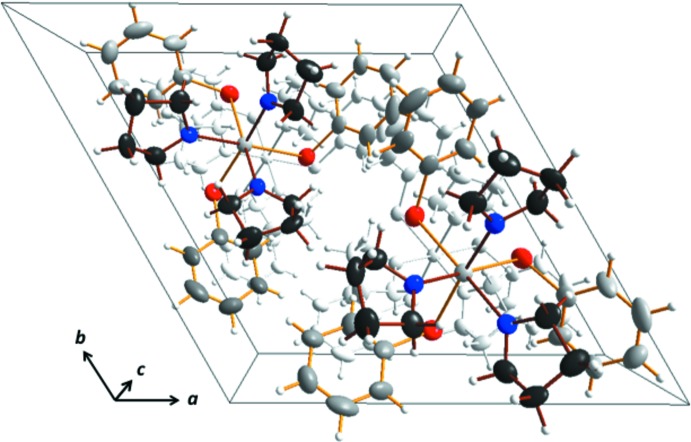
A perspective view along the c axis of the crystal packing of the title compound.

Click here for additional data file.x y z y x y z x y x z y x z x y y z x x y z . DOI: 10.1107/S1600536814023733/su5009fig1.tif
The mol­ecular structure of the title compound, with atom labelling. Displacement ellipsoids are drawn at the 50% probability level. Only the more abundant orientation of the disordered THF ligands is shown (symmetry codes: (i) *x*, *y*, *z*; (ii) −*y*, *x*-*y*, *z*; (iii) −*x* + *y*, −*x*, *z*; (iv) *y*, *x*, *z* + 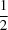; (v) *x*-*y*, −*y*, *z* + 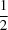; (vi) −*x*, −*x* + *y*, *z* + 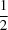.

c . DOI: 10.1107/S1600536814023733/su5009fig2.tif
A perspective view along the *c* axis of the crystal packing of the title compound.

CCDC reference: 1031290

Additional supporting information:  crystallographic information; 3D view; checkCIF report

## supplementary crystallographic information

### S1. Synthesis and crystallization

The title compound was synthesized by the literature procedure [Lee *et al.* (1998)]. The crystals were obtained from THF at 255 K.

### S2. Refinement

H atoms were positioned geometrically and refined using a riding model with C—H = 0.95 - 0.99 Å and with *U*_iso_(H) = 1.2*U*_eq_(C). The crystal is a non-merohedral with twin law of -1 0 0, 0 -1 0, 0 0 1 and a ratio of 0.65:0.35. The THF ligands show positional disorder with an occupancy ratio of 0.79 (3):0.21 (3).

### Crystal data

**Table:**

[Tm(C_6_H_5_Se)_3_(C_4_H_8_O)_3_]	*D*_x_ = 1.782 Mg m^−^^3^
*M**_r_* = 853.42	Mo *K*α radiation, λ = 0.71073 Å
Trigonal, *P*31*c*	Cell parameters from 2053 reflections
*a* = 15.277 (2) Å	θ = 3.1–28.1°
*c* = 7.8708 (16) Å	µ = 6.25 mm^−^^1^
*V* = 1590.9 (6) Å^3^	*T* = 120 K
*Z* = 2	Block, pale yellow-green
*F*(000) = 828	0.40 × 0.20 × 0.10 mm

### Data collection

**Table:**

Bruker–Nonius KappaCCD diffractometer	2053 reflections with *I* > 2σ(*I*)
φ scans, and ω scans with κ offsets	*R*_int_ = 0.053
Absorption correction: multi-scan (*XPREP* in *SHELXTL*; Sheldrick, 2008)	θ_max_ = 28.1°, θ_min_ = 3.1°
*T*_min_ = 0.189, *T*_max_ = 0.574	*h* = −16→20
5660 measured reflections	*k* = −18→14
2151 independent reflections	*l* = −9→10

### Refinement

**Table:**

Refinement on *F*^2^	H-atom parameters constrained
Least-squares matrix: full	*w* = 1/[σ^2^(*F*_o_^2^) + 8.380*P*] where *P* = (*F*_o_^2^ + 2*F*_c_^2^)/3
*R*[*F*^2^ > 2σ(*F*^2^)] = 0.038	(Δ/σ)_max_ < 0.001
*wR*(*F*^2^) = 0.087	Δρ_max_ = 0.76 e Å^−^^3^
*S* = 1.10	Δρ_min_ = −1.43 e Å^−^^3^
2151 reflections	Extinction correction: *SHELXL2013* (Sheldrick, 20008), Fc^*^=kFc[1+0.001xFc^2^λ^3^/sin(2θ)]^-1/4^
127 parameters	Extinction coefficient: 0.0089 (14)
9 restraints	Absolute structure: Flack *x* determined using 812 quotients [(*I*^+^)-(*I*^-^)]/[(*I*^+^)+(*I*^-^)] (Parsons & Flack, 2004)
Hydrogen site location: inferred from neighbouring sites	Absolute structure parameter: −0.03 (3)

### Special details

**Table:**

Geometry. All e.s.d.'s (except the e.s.d. in the dihedral angle between two l.s. planes) are estimated using the full covariance matrix. The cell e.s.d.'s are taken into account individually in the estimation of e.s.d.'s in distances, angles and torsion angles; correlations between e.s.d.'s in cell parameters are only used when they are defined by crystal symmetry. An approximate (isotropic) treatment of cell e.s.d.'s is used for estimating e.s.d.'s involving l.s. planes.
Refinement. Refined as a 2-component twin.

### Fractional atomic coordinates and isotropic or equivalent isotropic displacement parameters (Å^2^)

**Table:**

	*x*	*y*	*z*	*U*_iso_*/*U*_eq_	Occ. (<1)
Tm1	0.6667	0.3333	0.08579 (18)	0.0326 (3)	
Se1	0.63991 (14)	0.46785 (13)	−0.11260 (16)	0.0456 (4)	
O1	0.7727 (7)	0.4562 (7)	0.2822 (12)	0.034 (2)	
C1	0.7309 (12)	0.5979 (12)	−0.0110 (17)	0.040 (4)	
C2	0.6951 (13)	0.6510 (11)	0.081 (3)	0.055 (4)	
H2	0.6247	0.6222	0.1023	0.066*	
C3	0.7629 (18)	0.7463 (15)	0.142 (2)	0.069 (6)	
H3	0.7377	0.7829	0.2025	0.082*	
C4	0.8656 (14)	0.7899 (15)	0.119 (2)	0.061 (6)	
H4	0.9107	0.8545	0.1655	0.074*	
C5	0.9021 (15)	0.7375 (14)	0.026 (2)	0.056 (5)	
H5	0.9725	0.7669	0.0057	0.067*	
C6	0.8341 (15)	0.6407 (12)	−0.038 (2)	0.051 (5)	
H6	0.8591	0.6043	−0.0998	0.061*	
C7A	0.7418 (19)	0.5034 (16)	0.406 (2)	0.044 (5)	0.79 (3)
H7AA	0.6992	0.4534	0.4932	0.052*	0.79 (3)
H7BA	0.7017	0.5302	0.3514	0.052*	0.79 (3)
C8A	0.8335 (15)	0.587 (2)	0.487 (3)	0.061 (8)	0.79 (3)
H8AA	0.8218	0.5946	0.6082	0.074*	0.79 (3)
H8BA	0.8556	0.6521	0.4276	0.074*	0.79 (3)
C9A	0.9087 (15)	0.5529 (15)	0.465 (2)	0.044 (6)	0.79 (3)
H9AA	0.9009	0.5034	0.5539	0.053*	0.79 (3)
H9BA	0.9789	0.6104	0.4666	0.053*	0.79 (3)
C10A	0.879 (2)	0.505 (3)	0.293 (4)	0.045 (7)	0.79 (3)
H0AA	0.9104	0.5568	0.2032	0.055*	0.79 (3)
H0BA	0.9021	0.4551	0.2782	0.055*	0.79 (3)
C7B	0.743 (9)	0.484 (8)	0.454 (9)	0.044 (5)	0.21 (3)
H7AB	0.7077	0.5231	0.4379	0.052*	0.21 (3)
H7BB	0.7010	0.4239	0.5246	0.052*	0.21 (3)
C8B	0.850 (6)	0.551 (8)	0.532 (12)	0.061 (8)	0.21 (3)
H8AB	0.8521	0.6075	0.5967	0.074*	0.21 (3)
H8BB	0.8655	0.5099	0.6107	0.074*	0.21 (3)
C9B	0.928 (5)	0.592 (6)	0.386 (9)	0.044 (6)	0.21 (3)
H9AB	0.9961	0.6094	0.4276	0.053*	0.21 (3)
H9BB	0.9320	0.6533	0.3350	0.053*	0.21 (3)
C10B	0.888 (7)	0.505 (13)	0.26 (2)	0.045 (7)	0.21 (3)
H0AB	0.9122	0.4568	0.2814	0.055*	0.21 (3)
H0BB	0.9084	0.5304	0.1378	0.055*	0.21 (3)

### Atomic displacement parameters (Å^2^)

**Table:**

	*U*^11^	*U*^22^	*U*^33^	*U*^12^	*U*^13^	*U*^23^
Tm1	0.0385 (3)	0.0385 (3)	0.0206 (4)	0.01927 (16)	0.000	0.000
Se1	0.0548 (9)	0.0551 (10)	0.0316 (7)	0.0311 (9)	−0.0015 (8)	0.0096 (8)
O1	0.036 (5)	0.041 (6)	0.024 (4)	0.020 (5)	0.007 (4)	0.001 (4)
C1	0.054 (11)	0.045 (8)	0.028 (8)	0.031 (8)	0.005 (7)	0.017 (6)
C2	0.070 (10)	0.056 (10)	0.052 (9)	0.041 (9)	0.036 (12)	0.019 (11)
C3	0.13 (2)	0.072 (13)	0.042 (9)	0.077 (15)	0.016 (11)	0.012 (9)
C4	0.066 (12)	0.045 (9)	0.054 (13)	0.013 (8)	−0.005 (8)	−0.007 (8)
C5	0.061 (11)	0.045 (9)	0.069 (12)	0.033 (9)	0.012 (8)	0.006 (9)
C6	0.045 (10)	0.053 (9)	0.043 (8)	0.016 (9)	−0.009 (8)	−0.002 (6)
C7A	0.059 (11)	0.048 (11)	0.014 (9)	0.020 (10)	0.006 (11)	0.010 (8)
C8A	0.064 (14)	0.062 (18)	0.038 (14)	0.016 (12)	−0.003 (10)	−0.017 (12)
C9A	0.040 (11)	0.043 (12)	0.026 (10)	0.004 (10)	0.001 (9)	0.011 (8)
C10A	0.053 (12)	0.060 (11)	0.03 (2)	0.034 (10)	−0.001 (11)	0.003 (13)
C7B	0.059 (11)	0.048 (11)	0.014 (9)	0.020 (10)	0.006 (11)	0.010 (8)
C8B	0.064 (14)	0.062 (18)	0.038 (14)	0.016 (12)	−0.003 (10)	−0.017 (12)
C9B	0.040 (11)	0.043 (12)	0.026 (10)	0.004 (10)	0.001 (9)	0.011 (8)
C10B	0.053 (12)	0.060 (11)	0.03 (2)	0.034 (10)	−0.001 (11)	0.003 (13)

### Geometric parameters (Å, º)

**Table:**

Tm1—O1^i^	2.345 (10)	C7A—C8A	1.49 (2)
Tm1—O1	2.345 (10)	C7A—H7AA	0.9900
Tm1—O1^ii^	2.345 (10)	C7A—H7BA	0.9900
Tm1—Se1^i^	2.7692 (17)	C8A—C9A	1.49 (2)
Tm1—Se1^ii^	2.7692 (17)	C8A—H8AA	0.9900
Tm1—Se1	2.7692 (17)	C8A—H8BA	0.9900
Se1—C1	1.938 (18)	C9A—C10A	1.50 (2)
O1—C10A	1.41 (3)	C9A—H9AA	0.9900
O1—C7A	1.42 (3)	C9A—H9BA	0.9900
O1—C10B	1.55 (10)	C10A—H0AA	0.9900
O1—C7B	1.56 (10)	C10A—H0BA	0.9900
C1—C2	1.39 (2)	C7B—C8B	1.55 (9)
C1—C6	1.39 (3)	C7B—H7AB	0.9900
C2—C3	1.38 (3)	C7B—H7BB	0.9900
C2—H2	0.9500	C8B—C9B	1.55 (9)
C3—C4	1.38 (3)	C8B—H8AB	0.9900
C3—H3	0.9500	C8B—H8BB	0.9900
C4—C5	1.39 (2)	C9B—C10B	1.55 (9)
C4—H4	0.9500	C9B—H9AB	0.9900
C5—C6	1.41 (2)	C9B—H9BB	0.9900
C5—H5	0.9500	C10B—H0AB	0.9900
C6—H6	0.9500	C10B—H0BB	0.9900
			
O1^i^—Tm1—O1	81.2 (3)	C8A—C7A—H7BA	110.0
O1^i^—Tm1—O1^ii^	81.2 (3)	H7AA—C7A—H7BA	108.3
O1—Tm1—O1^ii^	81.2 (3)	C7A—C8A—C9A	102 (2)
O1^i^—Tm1—Se1^i^	94.4 (2)	C7A—C8A—H8AA	111.4
O1—Tm1—Se1^i^	92.6 (2)	C9A—C8A—H8AA	111.4
O1^ii^—Tm1—Se1^i^	173.0 (2)	C7A—C8A—H8BA	111.4
O1^i^—Tm1—Se1^ii^	92.6 (2)	C9A—C8A—H8BA	111.4
O1—Tm1—Se1^ii^	173.0 (2)	H8AA—C8A—H8BA	109.2
O1^ii^—Tm1—Se1^ii^	94.4 (2)	C8A—C9A—C10A	100 (2)
Se1^i^—Tm1—Se1^ii^	91.32 (6)	C8A—C9A—H9AA	111.7
O1^i^—Tm1—Se1	173.0 (2)	C10A—C9A—H9AA	111.7
O1—Tm1—Se1	94.4 (2)	C8A—C9A—H9BA	111.7
O1^ii^—Tm1—Se1	92.6 (2)	C10A—C9A—H9BA	111.7
Se1^i^—Tm1—Se1	91.32 (6)	H9AA—C9A—H9BA	109.5
Se1^ii^—Tm1—Se1	91.32 (6)	O1—C10A—C9A	107.3 (17)
C1—Se1—Tm1	103.4 (4)	O1—C10A—H0AA	110.3
C10A—O1—C7A	106.2 (19)	C9A—C10A—H0AA	110.3
C10B—O1—C7B	114 (7)	O1—C10A—H0BA	110.3
C10A—O1—Tm1	127.8 (12)	C9A—C10A—H0BA	110.3
C7A—O1—Tm1	125.8 (11)	H0AA—C10A—H0BA	108.5
C10B—O1—Tm1	117 (4)	C8B—C7B—O1	100 (7)
C7B—O1—Tm1	128 (4)	C8B—C7B—H7AB	111.8
C2—C1—C6	119.4 (16)	O1—C7B—H7AB	111.8
C2—C1—Se1	121.6 (13)	C8B—C7B—H7BB	111.8
C6—C1—Se1	118.9 (12)	O1—C7B—H7BB	111.8
C3—C2—C1	119.3 (16)	H7AB—C7B—H7BB	109.5
C3—C2—H2	120.3	C9B—C8B—C7B	109 (7)
C1—C2—H2	120.3	C9B—C8B—H8AB	110.0
C4—C3—C2	122.4 (17)	C7B—C8B—H8AB	110.0
C4—C3—H3	118.8	C9B—C8B—H8BB	110.0
C2—C3—H3	118.8	C7B—C8B—H8BB	110.0
C3—C4—C5	118.7 (17)	H8AB—C8B—H8BB	108.3
C3—C4—H4	120.7	C8B—C9B—C10B	105 (7)
C5—C4—H4	120.7	C8B—C9B—H9AB	110.9
C4—C5—C6	119.8 (17)	C10B—C9B—H9AB	110.9
C4—C5—H5	120.1	C8B—C9B—H9BB	110.9
C6—C5—H5	120.1	C10B—C9B—H9BB	110.9
C1—C6—C5	120.4 (17)	H9AB—C9B—H9BB	108.9
C1—C6—H6	119.8	C9B—C10B—O1	101 (7)
C5—C6—H6	119.8	C9B—C10B—H0AB	111.7
O1—C7A—C8A	108.6 (19)	O1—C10B—H0AB	111.7
O1—C7A—H7AA	110.0	C9B—C10B—H0BB	111.7
C8A—C7A—H7AA	110.0	O1—C10B—H0BB	111.7
O1—C7A—H7BA	110.0	H0AB—C10B—H0BB	109.4
			
C6—C1—C2—C3	1 (3)	C10B—O1—C10A—C9A	170 (51)
Se1—C1—C2—C3	−176.1 (14)	C7B—O1—C10A—C9A	3 (5)
C1—C2—C3—C4	−2 (3)	Tm1—O1—C10A—C9A	−163.7 (15)
C2—C3—C4—C5	2 (3)	C8A—C9A—C10A—O1	−37 (4)
C3—C4—C5—C6	−2 (3)	C10A—O1—C7B—C8B	1 (7)
C2—C1—C6—C5	−1 (2)	C7A—O1—C7B—C8B	−103 (15)
Se1—C1—C6—C5	176.5 (12)	C10B—O1—C7B—C8B	−2 (11)
C4—C5—C6—C1	1 (3)	Tm1—O1—C7B—C8B	168 (5)
C10A—O1—C7A—C8A	3 (3)	O1—C7B—C8B—C9B	23 (10)
C10B—O1—C7A—C8A	−3 (10)	C7B—C8B—C9B—C10B	−37 (14)
C7B—O1—C7A—C8A	85 (15)	C8B—C9B—C10B—O1	32 (14)
Tm1—O1—C7A—C8A	−171.5 (13)	C10A—O1—C10B—C9B	−33 (38)
O1—C7A—C8A—C9A	−27 (2)	C7A—O1—C10B—C9B	1 (15)
C7A—C8A—C9A—C10A	37 (3)	C7B—O1—C10B—C9B	−19 (14)
C7A—O1—C10A—C9A	21 (4)	Tm1—O1—C10B—C9B	170 (7)

Symmetry codes: (i) −*x*+*y*+1, −*x*+1, *z*; (ii) −*y*+1, *x*−*y*, *z*.
