# Supplementary figures and images for: Crystal structure of tris­(phenyl­seleno­lato-κSe)tris­(tetra­hydro­furan-κO)thulium(III)
Source: Acta Crystallogr Sect E Struct Rep Online. 2014 Oct 31;70(Pt 11):m389. doi: 10.1107/S1600536814023733 (PMC4257276; doi:10.1107/S1600536814023733)

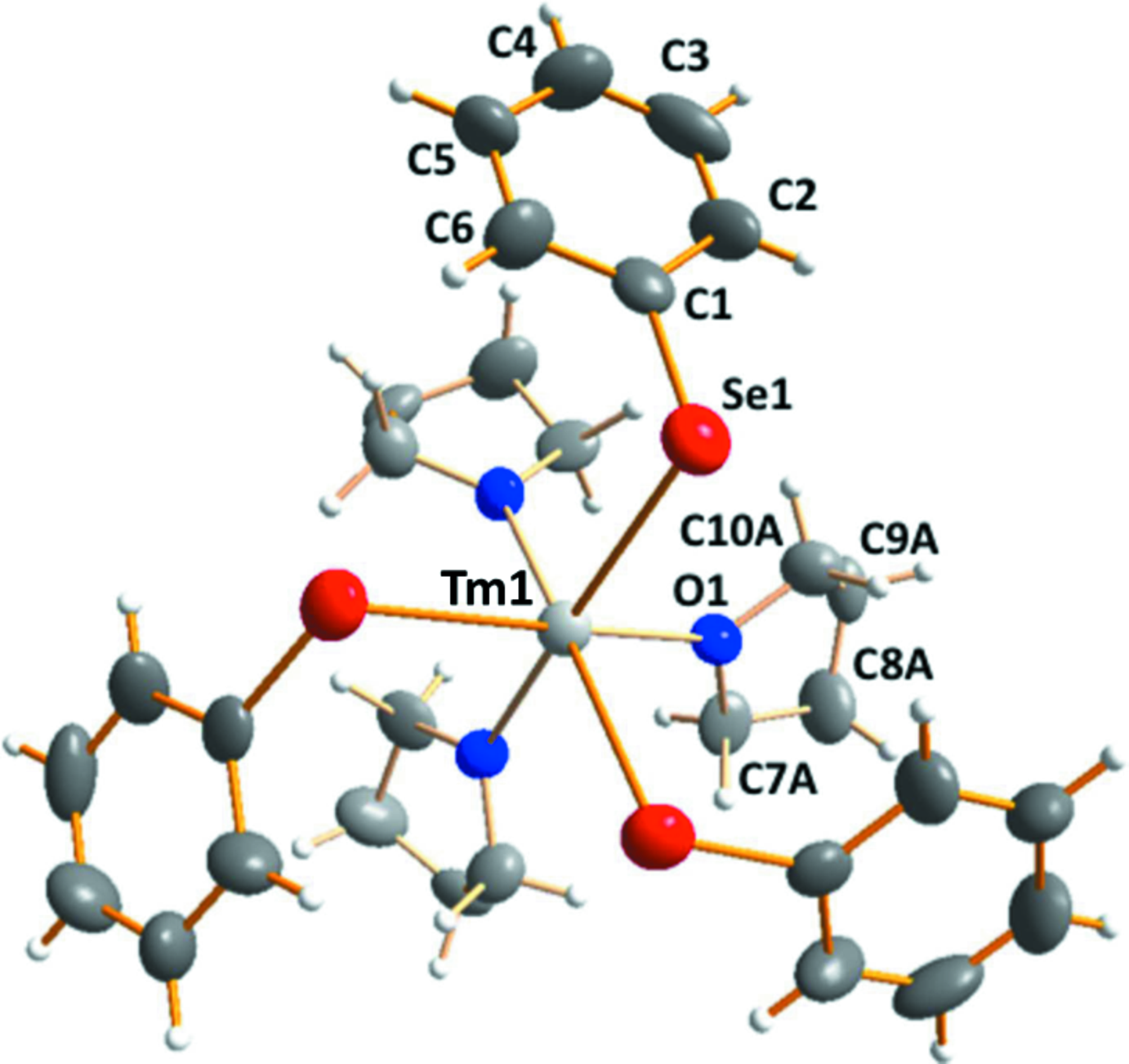

Supplement: Supplementary file 3 [file e-70-0m389-fig1.tif]

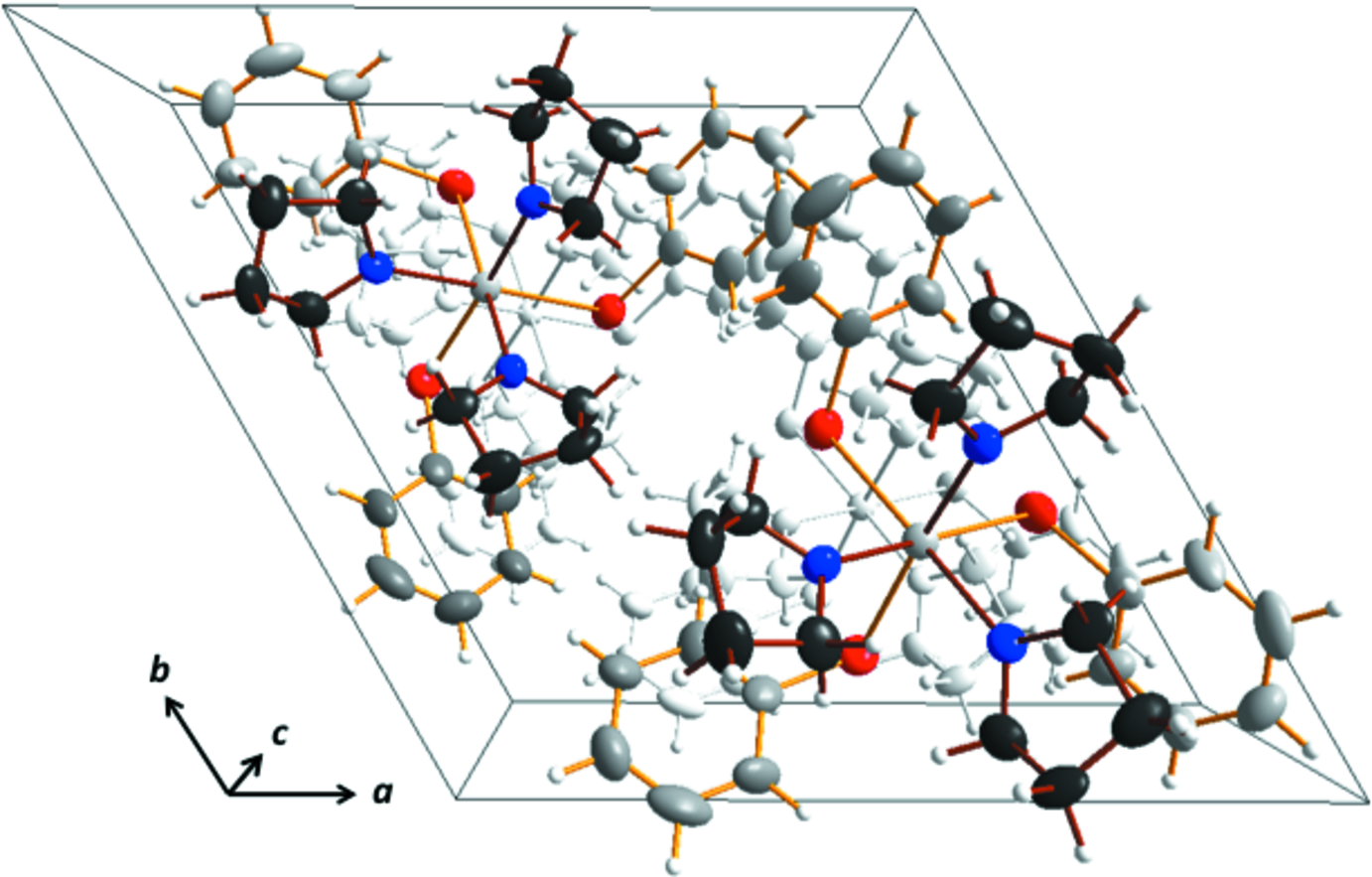

Supplement: Supplementary file 4 [file e-70-0m389-fig2.tif]
